# Sleep disorders related to nutrition and digestive diseases: a neglected clinical condition

**DOI:** 10.7150/ijms.45512

**Published:** 2021-01-01

**Authors:** Filippo Vernia, Mirko Di Ruscio, Antonio Ciccone, Angelo Viscido, Giuseppe Frieri, Gianpiero Stefanelli, Giovanni Latella

**Affiliations:** Division of Gastroenterology, Hepatology and Nutrition, Department of Life, Health, and Environmental Sciences, University of L'Aquila, Piazza S. Tommasi, 1- Coppito, 67100 L'Aquila, Italy.

**Keywords:** sleep disorders, circadian rhythm, diet, nutrition, gastrointestinal disease, digestive diseases

## Abstract

Sleep disturbances often result from inappropriate lifestyles, incorrect dietary habits, and/or digestive diseases. This clinical condition, however, has not been sufficiently explored in this area. Several studies have linked the circadian timing system to the physiology of metabolism control mechanisms, energy balance regulation, and nutrition. Sleep disturbances supposedly trigger digestive disorders or conversely represent specific clinical manifestation of gastrointestinal (GI) diseases. Poor sleep may worsen the symptoms of GI disorders, affecting the quality of life. Conversely, short sleep may influence dietary choices, as well as meal timing, and the circadian system drives temporal changes in metabolic patterns. Emerging evidence suggests that patients with inappropriate dietary habits and chronic digestive disorders often sleep less and show lower sleep efficiency, compared with healthy individuals. Sleep disturbances may thus represent a primary symptom of digestive diseases. Further controlled trials are needed to fully understand the relationship between sleep disturbances, dietary habits, and GI disorders. It may be also anticipated that the evaluation of sleep quality may prove useful to drive positive interventions and improve the quality of life in a proportion of patients.

This review summarizes data linking sleep disorders with diet and a series of disease including gastro-esophageal reflux disease, peptic disease, functional gastrointestinal disorders, inflammatory bowel diseases, gut microbiota alterations, liver and pancreatic diseases, and obesity. The evidence supporting the complex interplay between sleep dysfunction, nutrition, and digestive diseases is discussed.

## Introduction

Sleep disorders affect many individuals worldwide and their prevalence is increasing. It has been estimated that up to 70 million people in the US and 45 million in Europe suffer from a chronic sleep disorder that adversely affects health and quality of life (QoL) 1,2.

Sleep disorders are considered as risk factors for mortality, and two meta-analyses have reported an increased mortality rate among short sleepers 3,4. Pooled analyses of the work by Cappuccio et al. indicates that short sleepers (commonly defined as < 7 h per night, by fewer authors as < 5 h per night) have a 12% greater risk, and long sleepers (> 8 or 9 h per night) a 30% greater risk of dying than those sleeping 7 to 8 h per night 3. Similar results have been obtained by Ge et al., reporting assessing that difficulty in falling asleep (DFA) and non-restorative sleep (NRS) were associated with an increased risk of all-cause mortality (DFA: HR = 1.13, 95%: CI 1.03 to 1.23, *p* = 0.009; NRS: HR = 1.23, 95%CI: 1.07 to 1.42, *p* = 0.003) 4. Sleep disorders are estimated to favor drowsiness and represent the underlying cause of 15-20% of road accidents 5. Similarly, 13% of work injuries can be attributed to sleep problems 6.

A precise evaluation of costs deriving from sleep disorders is difficult. Their total cost in Europe was estimated at €35.4 billion in 2010 2. According to the Australian Sleep Health Foundation, the direct cost associated with the three commonest sleep disorders, obstructive sleep apnoea syndrome (OSAS), primary insomnia, and restless leg syndrome (RLS) was $818 million and the indirect costs exceeded $4.3 billion in 2010 7. Similar findings have been reported by the Italian Ministry of Health, which put the direct and indirect cost of OSAS in 2014 at €2,9 billion 8.

Sleep disorders are not limited to western countries but are increasingly frequent in developing world. According to a recent study of Asian and African patients, 16.6% of participants had severe nocturnal sleep problems 9.

The International Classification of Sleep Disorders (ICSD) is the key reference for the diagnosis of sleep disorders, classified in seven major categories: insomnia disorders, sleep-related breathing disorders, central disorders of hypersomnolence, circadian rhythm sleep-wake disorders, sleep-related movement disorders, parasomnias, and other sleep disorders 10,11.

**Figure 1:**
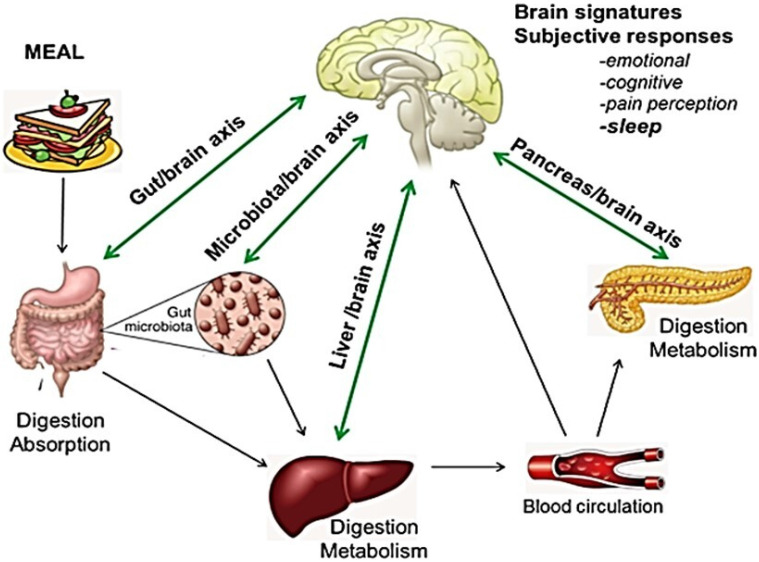
** Bidirectional interaction between GI tract, liver, pancreas, and brain: food and gut-brain connection.** The central nervous system (CNS) plays a role in regulating function and homeostasis of the gastrointestinal tract. Nutrients affect the production of hormones, including growth hormone, prolactin, testosterone, melatonin, and serotonin, all playing a role in the regulation of the circadian rhythms and brain function. Similarly, digestive hormones and enteroendocrine secretions (GLP-1, GIP, serotonin, substance P and peptide YY) also activate visceral afferent endings. Pancreatic endocrine secretion, through glucose homeostasis, insulin-resistance and GLP-1 activity is also implicated in the crosstalk between gut and neurological function. Impairment of liver detoxifying processes results in increased concentrations of false neurotransmitters and CNS-acting toxins. The gut flora also influences the CNS, regulating the architecture of sleep, stress reactivity and behavior. Bacterial metabolic products and inflammatory cytokines have been proposed as possible mediators. Adapted from E. M. Candeias et al. World J Diabetes 2015;6:807-827.

Sleep disorders are related to a variety of causes that prevent the straightforward identification of the primary mechanism involved in individual patients, but are often related to poor diet, inappropriate lifestyle and dietary habits, and/or digestive diseases. Indeed, a close relationship is widely documented between brain and nutrition/metabolism, as well as between brain and digestive organs. (**Fig. 1**) 12-15. However, little is known about the clinical impact of this emerging issue.

The aim of this review is to discuss and summarize the current evidence on the role of dietary habits, and GI conditions, in sleep disorders, identifying therapeutic gaps and opportunities. Limitations of recently performed studies, will underline the need for additional, multi-specialist, clinical trials to elucidate the complex bidirectional interaction between sleep disorders and GI tract diseases.

## Materials and methods

A systematic electronic search of the scientific literature up to January 2020 was performed using Medline, EMBASE, Web of Science, Scopus, and the Cochrane Library. The search strategy used a combination of Medical Subject Headings (MeSH) and keywords as follows: “sleep”, “sleep disorders”, “circadian clock”, “circadian rhythm”, “arousal”, “awakening”, “diet”, “nutrition”, “nutrients”, “energy balance”, “metabolism”, “metabolic syndrome”, “eating habits”, “food intolerances”, “lactose intolerance”, “gluten intolerance”, “food allergies”, “gastrointestinal disease”, “gastro-esophageal reflux disease”, “peptic disease”, “ulcer”, “nausea”, “vomiting”, “functional dyspepsia”, “eructation”, “rumination”, “irritable bowel syndrome”, “functional flatulence”, “constipation”, “diarrhea”, “incontinence”, “anorectal pain”, “inflammatory bowel disease”, “IBD”, “coeliac disease”, “gut microbiome”, “gut microbiota”, “dysbiosis”, “gut-brain axis”, “liver diseases”, “cirrhosis”, “hepatic encephalopathy”, “chronic hepatitis”, “acute hepatitis”, “non-alcoholic fatty liver disease”, “non-alcoholic steatohepatitis”, “pancreatic diseases”, “chronic pancreatitis”, “acute pancreatitis”, “pancreatic insufficiency”, “non-alcoholic fatty pancreatic disease”, “overweight”, “obesity”, “obstructive sleep apnoea”. Relevant articles were identified by screening the abstract; additional studies were identified by a manual search of the reference list of studies and review articles. The great majority of articles reported in the present review did not use the ICSD-3 classification, minimizing the possibility to categorize the interaction between different conditions and the mechanisms of sleeping disorders.

## Nutrition and sleep disorders

A healthy diet and lifestyle, and correct eating habits contribute to psychological and physical wellbeing, sleep included, whereas an inappropriate diet and limited physical activity impair the quality of sleep 16,17.

Eating too quickly or skipping meals, overabundant meals, irregular mealtimes, and poor food quality, all are dietary causes of sleep disorders. Spicy food, stimulants, and adverse food reactions (intolerances and food allergies) may be additional factors.

The eating behavior affects quality and quantity of sleep in humans 18. A consistent association between short sleep and a high total energy intake has also been described 19. Nutrients may affect the production of hormones, including growth hormone, prolactin, testosterone, melatonin, and serotonin, all of which play a role in regulating the circadian clock. Nutrients favoring or inhibiting their release influence the quality of sleep, whereas foods acting on tryptophan availability or the synthesis of serotonin and melatonin promote sleep 20. Some vitamins (B1 and B6) also induce production and release of melatonin and serotonin 21, 22.

Stimulants, like coffee, cola beverages, spices, tea, and chocolate may also adversely affect sleep 23. Alcohol, one of the most powerful depressants, alters the circadian clock and worsens the quality of sleep 24. Spicy and hot foods are associated with insomnia 25.

The timing of meals, especially frequency and regularity of snacks, desynchronize circadian rhythm, affect metabolism, and favor obesity 26, 27. This is consistent with the role of the circadian clock in regulating the release of hormones and neurotransmitters involved in sleep control.

Skipping meals and eating a single, large evening meal is increasingly common in industrialized countries. However, shifting the main calorie intake to the end of the day adversely affects digestion and makes sleep difficult due to GI symptoms such as gastro-esophageal reflux (GERD), dyspepsia, and nausea; more so if the meal is abundant and fat-rich 28. Conversely, the role of carbohydrates on sleep patterns is still debated 29, 30 and the weight of carbohydrates versus caloric load is controversial.

Stress is important in influencing eating patterns, possibly through alterations in the hypothalamic-pituitary-adrenal axis favoring the consumption of junk food (high in fat and refined sugar) 31.

Sedentary lifestyle and short sleep time are associated with poor eating habits, and favor high energy intake 32-34. Limited physical activity during the day has been related to food consumption at night, which in turn promotes the metabolic syndrome and obesity and, again, adversely affects the circadian clock 35.

Food allergies and intolerances, involving lactose and gluten (coeliac disease and gluten sensitivity), also have a negative influence on sleep patterns 36. The presence in food of amino acids such as phenylalanine, histamine, and tyramine, promotes production and release of adrenaline, noradrenaline, and other stimulant neurotransmitters, may impair sleep.

According to some authors, the relationship between meals and sleep is reciprocal, since the circadian system drives temporal changes in metabolic patterns while changes in metabolic or nutritional status alter the circadian rhythm 37.

The release of some hormones involved in metabolism - e.g. glucagon, insulin, ghrelin, leptin, and corticosterone - is associated with a well-set circadian rhythm 38. Sleep disturbances alter the energy balance and may induce weight gain 39, obesity, and type 2 diabetes 40,41. The underlying mechanisms are largely unknown 42, but several lines of evidence link the circadian timing system to the physiology of metabolism and nutrition 43,44. Changes in neuroendocrine system activity are the main mediators of insufficient sleep, by increasing appetite, enhancing sensitivity to food stimuli, and favoring high energy intake 45.

Poor sleep is related to changes in the circulating concentration of melatonin, cortisol, ghrelin, and leptin 46-49. Other examples include daily fluctuations of glucose levels, insulin sensitivity, and postprandial response 37,50. Thus, the disruption of the circadian rhythm and sleep loss may help inducing the metabolic syndrome 51.

Ghrelin acts as a neuropeptide and participates in sleep-wake regulation. Systemic ghrelin infusion promotes non-REM sleep in males, both humans and mice, but not in healthy women 52.

Brain histamine regulates arousal, circadian, and eating rhythms. H3 receptor, which triggers its effect on sleep, is among the treating targets for sleeping disturbances 53,54.

Sleep restriction has been associated with reduced concentrations of the satiety factor leptin, and increased concentrations of the hunger-promoting hormone ghrelin, altering their ability to signal the correct caloric requirement 48,49. The dynamic interplay between the digestive system and sleep is an excellent example of the brain-body interaction (Fig.1) 12-15. The hypothalamus, particularly the lateral hypothalamic area, regulates the energy balance and coordinates peripheral cues of energy status, and weight-affecting behaviors. Lateral hypothalamic neurons expressing neuropeptides like melanin-concentrating hormone and orexin/hypocretins, through distinct circuits, play an important role in regulating the ingestion of food, arousal, locomotor behavior, and autonomic functions 56. Orexins provide a link between energy homeostasis and vigilance state and are involved in the dopaminergic reward system 57,58. Mutation of orexin-producing genes induces an altered sleep phenotype in animal models. High activity of orexin-producing cells during wake periods and almost none during sleep has also been hypothesized to impair sleep 59.

Mounting evidence also suggests that sleep influences dietary choices. Individuals who sleep less are more likely to prefer energy-rich foods (e.g. fats and refined carbohydrates), eat fewer vegetables, and choose irregular eating patterns 60-63.

## Digestive diseases and sleep disorders

Sleep disturbances and sleep deprivation induce a variety of visceral disorders 64 but may also be the symptoms of other diseases. Patients with chronic disorders often report short sleep or show a lower sleep quality, alone or associated with other symptoms, when compared to healthy individuals. 65. Poor sleep may also worsen the subjective symptoms of the disorders, affecting QoL 66.

The GI diseases that most frequently impair sleep are acid-related (GERD, peptic disease), functional diseases, inflammatory GI disorders, liver diseases, overweight, and obesity.

### Gastro-esophageal reflux disease

GERD is characterized by pathological acid or non-acid reflux and is associated with a variety of disturbances that may affect the upper GI tract (regurgitation, heartburn, pain) and/or induce respiratory symptoms (hoarseness, dysphonia, chronic laryngitis, cough, asthma, and chronic bronchitis). Awakenings due to heartburn, dyspepsia, acid brash, coughing, or choking are a major cause of sleep disruption 66,67. There is strong evidence for a bidirectional relationship between GERD and sleep disturbances, since GERD symptoms cause difficulty falling asleep, sleep fragmentation, and early morning awakenings, while in turn sleep deprivation appears to induce esophageal hyperalgesia 68. Thus, GERD patients with sleep disturbances report more severe symptoms and a worse QoL than those without sleep disturbances 69. The association between GERD and sleep disturbances has been recently confirmed by two different trials on Australian and Japanese patients 70,71. Interestingly, a study by Hoshino reported that approximately one-third of the patients with GERD and sleep disturbance had non-acid reflux 72. In these patients, high prevalence of anxiety and depression has been reported, the latter being to some extent directly mediated by poor sleep 70,71.

An association between GERD and sleep apnea is frequently reported 73. Reflux patterns are different during arousal and sleep, due to delayed gastric emptying, reduced peristalsis, reduced salivary secretion and swallowing, and prolonged esophageal clearing time during sleep.

GERD may be overlooked when patients fail to report common signs and symptoms (regurgitation, heartburn, substernal pain), which the patient does not consider relevant in sleep disturbances. A recent meta-analysis suggests that treatment with proton pump inhibitors (PPIs) for GERD improves the quality of sleep 74. Nutritional and behavior modification may prove useful besides PPIs and prokinetics: avoid large/late-night meals may help preventing reflux 69. Avoiding going to bed soon after the evening meal, smoking, eating fat-rich or acid foods, mint, chocolate, coffee, tea, fizzy drinks may be helpful. Weight loss may be considered as well as raising the bed headrest by 15-20 cm, in the most severe cases.

### Peptic disease

Peptic disease is characterized by specific symptoms including epigastric pain, heartburn, a sense of hunger or satiety, nausea, and vomiting, associated to gastric or duodenal mucosal lesions. Gastric acid secretion plays a central role in the pathogenesis.

Patients with duodenal ulcers often wake up during the night due to heartburn and epigastric pain, relieved by food, whereas gastric ulcers are associated with earlier postprandial symptoms, making digestion hard and causing epigastric pain, resulting in difficulties in falling asleep. Pain awakening affects two-thirds of patients with duodenal ulcers and one-third of those with gastric ulcers 75. PPI treatment associated, if needed, with Helicobacter pylori eradication, improves sleep quality in patients with peptic disease 74. Dietary recommendations consist in reducing protein, salt and calcium consumption, which induce gastric acid secretion. This proves most useful in the early or acute stages of the disease 76. Patients with gastric ulcer should reduce lipids as well, due to the unfavorable effect on gastric emptying 77.

Longer sleep duration may play a protective role against the development of peptic ulcers 78 whereas the disruption of the circadian rhythm by sleep disturbances or shift work, may favor in peptic ulcer disease 79.

### Functional gastrointestinal disorders

Functional disorders involving the upper GI tract (functional dyspepsia, eructation, nausea, vomiting, and rumination), the small and large bowel (irritable bowel syndrome [IBS], functional flatulence, functional stipsis/constipation, functional diarrhea), and the anorectum (functional anorectal pain, incontinence and functional rectal outlet obstruction) are often associated with sleep disorders. Impaired sleep quality and chronic fatigue are frequently reported in these patients. Recent data from 3600 Chinese patients with functional gastrointestinal disorders, indicate that excessive daytime sleepiness is present as well 80.

Symptoms may be triggered or exacerbated by stress or by nutrients. Patients with functional dyspepsia, besides treatment with prokinetics and anti-secretory medications, should adopt specific dietary restrictions 81,82 of pro-secretory food including proteins, salt, and calcium. Lipids, slowing gastric emptying 83 should also be cut down in functional dyspepsia.

Sleep disturbances are well documented in IBS patients. Difficulty falling asleep, short sleep time, frequent arousals and awakenings, and non-restorative sleep are common 84,85. The association is confirmed by two large trials, carried out the general population and nurses, especially in the presence of depression 86,87. A recent meta-analysis including 36 studies with 63620 participants, reported a 37.6% prevalence of sleep disorders in IBS patients 88.

Although the underlying mechanisms are still unclear, decreased orexin signaling may play a role in the pathogenesis of the functional GI disorder in some patients suffering from sleep disturbances 89. Melatonin modulates GI motility and sensitivity in IBS patients, through still unidentified mechanisms 90 and exerts analgesic effects in patients with chronic pain (e.g. IBS) 91.

Abnormal sensitivity to some nutrients, including lactose, gluten, and fermentable oligosaccharides, disaccharides, monosaccharides and polyols (FODMAPs) have often been reported in functional intestinal disorders 92, but the role of negative expectations after ingesting some food should be ruled out 93.

In patients with suspected functional symptoms, primary or secondary motility disorders (diabetes, neurodegenerative diseases) favoring small intestinal bacterial overgrowth (SIBO) should be ruled out. This condition characterized by an abnormal bacterial fermentation in the small bowel may mimic functional symptoms as well as abdominal pain worsening 94 and sleep disruption 95. SIBO should always be considered in patients undergoing prolonged PPI use and profound acid inhibition 96.

### Inflammatory intestinal disorders and celiac disease

Chronic inflammatory bowel diseases (IBD) are held to be a risk factor for sleep, but the available data are inadequate and largely inconclusive.

Inflammatory cytokines such as tumor necrosis factor-α (TNF-α), interleukin (IL) -1, and IL-6 cause sleep disturbances, while sleep deprivation upregulates cytokines 97, particularly IL-1 and TNF-α. Clinical studies identified an association between sleep disturbances, disease activity, subclinical inflammation, and risk of IBD relapse 98-100. A recent trial reported that poor sleep quality, evaluated using the Pittsburgh Sleep Quality Index, was associated to the absence of mucosal healing (*P*<0.05) 101. An improvement in sleep and mood quality was observed in patients responding to anti-TNF-α or vedolizumab 102. Conversely, the association between IBD and sleep disorders has been questioned by a study objectively measuring sleep parameters by actigraphy, urine melatonin metabolites and two different sleep quality indexes 103. These data are in line with other reports suggesting the importance of IBS-like symptoms on the sleep quality of IBD patients in clinical remission 104.

Data regarding sleep impairment in coeliac disease are scarce and contradictory. Breathing disorders during sleep, ranging from primary snoring to obstructive sleep apnea, have been reported, with gas exchange abnormalities and sleep fragmentation 105. According to a recent study, coeliac patients are at high risk of poor sleep, both before and after diagnosis 106. Other data are conflicting, supporting 107 or failing to confirm sleep problems in coeliac disease 108. Similarly, pediatric coeliac patients less likely present with OSA-related symptoms as compared to healthy controls. Nonetheless, gluten-free diet induced significantly improvement in those who had symptoms 109.

### Gut microbiota alterations

The alterations of the gut microbiota, besides being responsible for several gastrointestinal disorders, seem to be involved in sleep disorders. The gut microbiome affects the brain through many mechanisms. Several metabolites such as short chain fatty acids, biliary acid metabolites, and neuroactive substances (gamma aminobutyric acid, tryptophan precursors and derivatives, serotonin, and catecholamines) produced by bacteria, as well as cytokines released during the immune response mediate microbiome-host interactions 110-112. The influence of these signals has been documented in animal models 113 and seems to play a role in the development of the nervous system 114.

**Figure 2:**
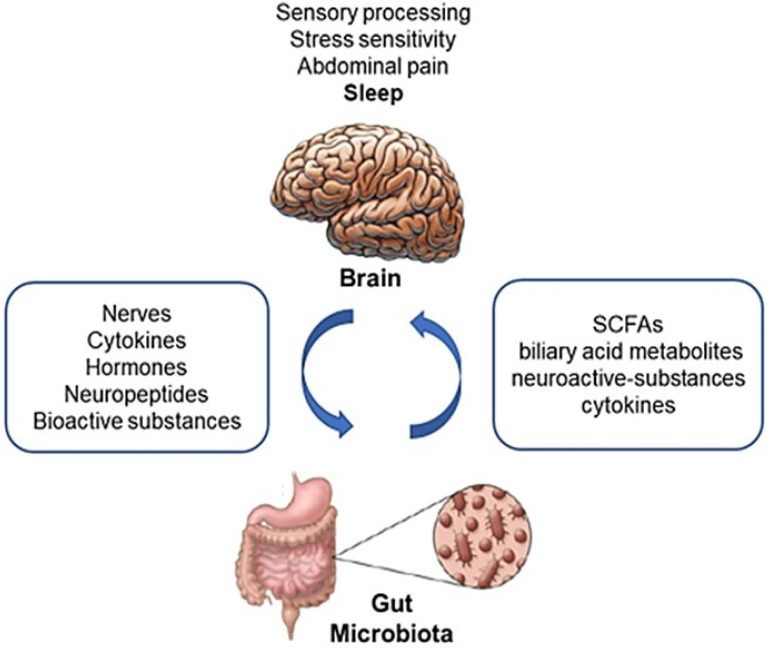
** Bidirectional interaction among the gut microbiota and the brain.** The gut microbiome affects the brain through a variety of mechanisms, producing or modifying several metabolites, neuroactive substances and cytokines. On the other hand, the Enteric Nervous System, receiving efferent information from the brain through autonomic neural connections and hormonal pathways, modulates gastrointestinal motor function, gastric secretion, intestinal absorption and secretion and gut-associated immune system, which all may impact on the composition and metabolism of the gut flora. SCFAs: Short Chain Fatty Acids. Adapted from Chen X, Protein Cell 2013;4:403-14.

The gut microbiota is involved in shaping the hypothalamic-pituitary-adrenal axis in relation to sleep and the stress reaction 15,95. It is also likely involved in the regulation of motivated behavior and emotions (**Fig. 2**) 115,116.

An extensive bidirectional communication network between the GI tract and the central nervous system (CNS), referred to as the “gut-brain axis”, has been widely investigated in the past decade (**Fig. 1**) 12-15. Recent studies linked psychiatric disorders, including depression, to changes in the microbiome 117,118. A large proportion of depressed patients present with changes in appetite and sleep (difficulty falling asleep, difficulty staying asleep, low sleep quality) that significantly impair their daily life. Data focused on sleep, however, are scarce 119.

Short sleep duration induces a physiological stress response, which in turn disrupts the normal balance of intestinal microbiota. People suffering from jetlag, shift work, early morning starts, and late bedtimes have altered intestinal microbial balance and dysbiosis 119,120.

Sleep and circadian rhythm disruption may alter the gut microbiota in humans, contributing to the development of metabolic disease 121. Sleep loss has been hypothesized to induce blood-brain barrier disruption in rodents. The underlying mechanism is unknown, but sleep loss has been postulated to induce low-grade systemic inflammation, releasing cytokines, chemokines, and acute-phase proteins 122.

Gut dysbiosis also seems to play a role in the development of OSA-induced hypertension 123.

These hypotheses have been questioned by a small study that aimed at exploring whether improving sleep duration is associated with changes in gut microbiota in chronically sleep-deprived individuals. The study failed to identify significant changes after two weeks of observation. The relationship between gut microbiota and sleep, if present, is thus still to be elucidated 124.

### Liver diseases

Sleep disorders may occur in both acute and chronic hepatitis but are more common in cirrhotic patients. A considerable proportion of patients with cirrhosis and acute or chronic liver failure suffer from insomnia, delayed sleep, and excessive daytime sleepiness. This association has been recently confirmed by a study on 341 patients with viral liver cirrhosis, reporting a significant elevation of the Pittsburgh Sleep Quality Index 125. Abnormal polysomnographic findings are also present 126. Sleep disturbances have been attributed to hepatic encephalopathy (HE). According to recent guidelines, HE can be subdivided into overt and covert HE 127. Covert HE, corresponding to mild or grade I disease according to West Haven criteria, affects up to 80% of cirrhosis patients 128. Sleep disturbances often are an early sign of HE, leading to daily sleepiness, increased risk of injuries, and impairing QoL 129,130. Covert HE is clinically underdiagnosed, unless specific tests are performed, such as the paper-pencil test or the Inhibitory Control test. No clear correlation has been found between sleep disturbances and HE, but some studies suggest impaired melatonin metabolism 131, since the response of melatonin to light is decreased in cirrhosis 132. The gut microbiota is involved in HE, as shown by the clinical improvement following lactulose or rifaximin, which reduce fermentation 133-135. Supplementation with oral branched chain amino acids has been reported to improve sleep in cirrhosis without encephalopathy, lessening the production of false neurotransmitters like octopamine by aromatic amino acids 136.

A recent meta-analysis involving 21 trials and 1420 participants suggested that probiotics may reduce the risk of developing overt HE and high plasma ammonia, thus improving QoL compared with placebo or no intervention 137.

Sleep disturbances have been reported in 60% of patients with chronic C virus (HCV) hepatitis 138,139. Moreover, complications of interferon α (used for the treatment of HCV) sometimes consist in sleep disorders 138,140.

**Figure 3:**
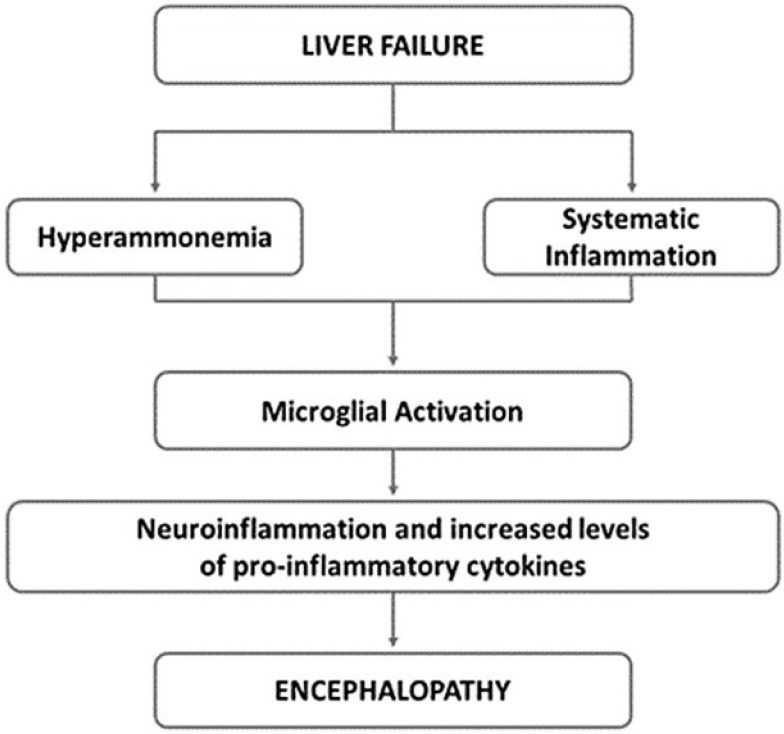
** Underlying mechanisms of hepatic encephalopathy.** Neuroinflammation is common in hepatic encephalopathy. It has been suggested that hyperammonaemia as well as systematic inflammation may lead to the activation of microglia. This activation induces to the local production of inflammatory mediators, that along with hyperammonaemia, negatively impact astrocyte function and contribute to the neurobehavioral deficits in hepatic encephalopathy. Adapted from Butterworth RF, Nat Rev Gastroenterol Hepatol 2013;10:522-528.

The neural and humoral communication pathways between liver and brain are not fully understood, but a role is played by inflammatory cytokines such as TNF-α, IL-1, and IL-6, which alter the concentration of central neurotransmitters (serotonin and corticotrophin-releasing hormone) (**Fig. 3**) 141,142.

An association with sleep disorders has been reported in chronic hepatitis B, but evidence is weak 143.

Liver damage and impairment of detoxification processes are associated with increased concentrations of false neurotransmitters and toxins, which may influence sleeping patterns. Hyperammonemia leads to cerebral dysfunction, involving a spectrum of neuropsychiatric and neurological symptoms including an inversion of the sleep-wake rhythm 144.

Primary biliary cirrhosis and Wilson disease are also associated with sleep disturbances, which possibly represent a negative prognostic factor for these patients 145.

**Figure 4:**
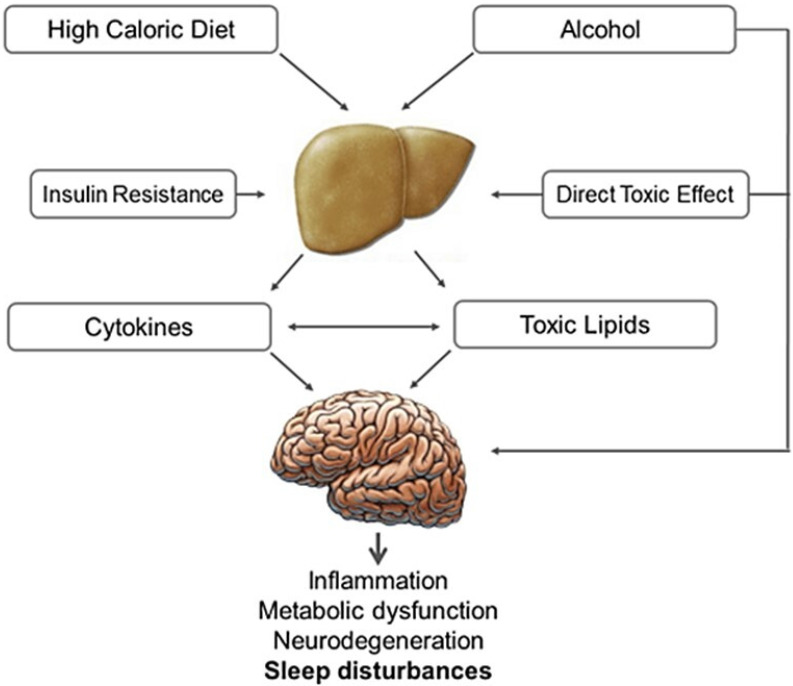
** Effect of alcoholic and non-alcoholic steatohepatitis on brain function.** High caloric and fat-rich diet and alcohol are the main causes of non-alcoholic and alcoholic steatohepatitis, which are proven to have a negative impact on brain function. Insulin plays an important role in both these conditions, compromising neural cell survival, metabolism and plasticity. In steatohepatitis the increased production of toxic lipids (e.g. ceramide), oxidative stress, as well as local cytokine production and activation, along with direct alcohol toxicity and depressant activity, may induce sleep disturbances, cognitive impairment and neurodegeneration.

Non-alcoholic fatty liver disease (NAFLD) and non-alcoholic steatohepatitis (NASH) are associated with insulin resistance, glucose intolerance and the metabolic syndrome 146. Sleep disorders in patients with steatohepatitis are likely related to impaired hepatocyte activity and impaired disposal of excess lipids. Alcohol has direct toxic effects on liver and CNS (**Fig. 4**). Several nuclear receptors expressed in the liver of patients with NAFLD and NASH influence the molecular clockwork throughout the day and may play a role in sleep disorders. 147 NASH and NAFLD are also related to OSA 148,149. A recent meta-analysis of 9 studies (2272 participants) reported that OSA is significantly correlated with steatosis, lobular inflammation, ballooning degeneration and fibrosis 150.

Pruritus is common in patients with chronic liver disease, more so in those with cholestatic liver diseases such as primary biliary cholangitis. Impaired sleep and low QoL often ensue 151.

The prevalence of pruritus in liver diseases varies from 5% in chronic hepatitis C to 70% in primary biliary cirrhosis 152. Increased concentrations of bile salts, histamine, serotonin, progesterone metabolites, and endogenous opioids are likely involved. However, a clear cause-effect link is lacking, as well as a correlation with the intensity of itching 153.

#### Pancreatic diseases

Pain due to chronic pancreatitis is associated with impaired cognitive function, anxiety, depressive symptoms, and sleep disturbances 154.

Sleep quality is somewhat reduced in patients with severe pancreatic insufficiency undergoing home parenteral nutrition compared with aged-matched controls. Overall, sleep quality is not affected by home parenteral infusion 155. OSA may be a risk factor for non-alcoholic fatty pancreatic disease, but the underlying mechanisms require clarification 156.

## Obesity

Overweight and obesity favor sleep disorders through the increased prevalence of GERD and NAFLD, as well as OSA.

OSA is a common sleep disorder characterized by partial or complete upper airway occlusion during sleep, involving intermittent cessation (apnea) or reduction (hypopnea) of airflow and dips in arterial oxygen saturation during sleep 157.

Obesity and OSA have a reciprocal relationship. The sleep disruption of OSA promotes behavioral, metabolic, and/or hormonal changes, favoring weight gain and/or difficulty in losing it. OSA is associated with a hormonal profile characterized by high leptin and ghrelin levels, which in turn promote excess energy intake 158.

A 10% gain in body weight is associated with a 50% increase in the probability of developing OSA. Conversely, weight loss leads to less severe OSA, sleep improvement, and further weight loss. A relationship between OSA, short sleep duration and weight gain has thus been postulated 39,159. Some evidence suggests that hypersomnolence is linked with obesity, also in the absence of sleep apnea 160.

## Medications

Sleep is adversely affected by a variety of medications, hampering respiration, exacerbating or causing apnoea, inducing awakenings, and disrupting physiological sleep patterns. Muscle relaxants, by reducing muscle tone, can worsen OSA, a condition in which sedatives and hypnotics are contraindicated 161,162. Alcohol and cigarette smoking (nicotinic acid) also display an unwanted myorelaxant effect 163. Chronic opioid use is associated with disordered breathing during sleep and central sleep apnea 164.

Selective serotonin reuptake inhibitors are involved in sleep disorders 165, due to the central role of the serotoninergic system in the modulation of sleep, eating, emotional status and pain 166.

## Conclusions

The link between diet, meal timing and sleep is reciprocal, as the circadian rhythm drives changes in the metabolic pattern, while modifications in the metabolic and nutritional status influence the circadian rhythm. Poor sleep is consistently related to changes in circulating melatonin, cortisol, ghrelin, and leptin, but the existence of additional mechanisms is likely.

Associations have also been found between short sleep duration, high total energy intake, and low-quality diet. Short sleepers often display irregular eating behaviors and take their main meal late in the day.

Sleep disturbances and sleep deprivation may either lead to, or worsen, visceral disorders but may in turn represent a symptom of disease. Chronic digestive diseases like GERD and peptic disease, functional and inflammatory GI disorders, liver diseases, often result in shorter, poor-quality sleep. Data, however, are mainly based on poor quality studies. Thus, adequately powered epidemiological studies and controlled trials focused on chronic short sleepers are needed to confirm causal relations. Sleep extension trials involving patients with GI disorders are needed as well to provide evidence on the relationship between GI disorders, diet and sleep duration and quality.

An integrated approach, involving sleep-specialists and gastroenterologists, using validated questionnaires and the ICSD-3 classification, is thus recommended to outline and categorize the specific sleep pattern impaired by, or involved in, GI disorders. A multi-specialist approach may indeed provide an insight on the complex bidirectional interaction between sleep disorders and GI tract diseases and, possibly, identify new therapeutic targets aimed at improving the quality of life of patients.

## Authors' Contributions

F.V., M.D.R., A.C., and G.S. performed the literature review, wrote the manuscript, and prepared the Figures; A.V and G.F. reviewed the manuscript and provided critical comments; G.L. suggested the topic of the review and supervised, wrote, and critically reviewed the manuscript. All authors read and approved the final draft of the manuscript.
